# Supplementary material for: Discovery of 2′,6-Bis(4-hydroxybenzyl)-2-acetylcyclohexanone, a Novel FtsZ Inhibitor
Source: Molecules. 2022 Oct 18;27(20):6993. doi: 10.3390/molecules27206993 (PMC9610434; doi:10.3390/molecules27206993)
Supplement: Supplementary file 1 [file molecules-27-06993-s001.zip › molecules-1977304-supplementary.pdf]

molecules

SUPPLEMENTARY MATERIALS

**Discovery of 2',6-bis(4-hydroxybenzyl)-2-acetylcyclohexanone, a novel FtsZ inhibitor**

Hsuan-Yu J Lin[1#], Rachana Rao Battaje[2#], Jinlong Tan[1], Munikumar Doddareddy[1], Hemendra Pal Singh Dhaked[2], Shalini Srivastava[2], Bryson A. Hawkins[1], Laith Mohammad Hilal Al-shdifat[1], David E. Hibbs[1], Dulal Panda[2,3*], Paul W. Groundwater[1*]

[1]School of Pharmacy, Faculty of Medicine and Health, University of Sydney, Sydney, New South Wales, Australia
[2]Department of Biosciences and Bioengineering, Indian Institute of Technology Bombay, Mumbai, India
[3]National Institute of Pharmaceutical Education and Research, S.A.S. Nagar Punjab 160062, India

**Supporting Information**

**X-Ray Crystallography**

X-Ray crystallographic data was acquired for (1) on an Agilent dual-beam SuperNova™ X-Ray diffractometer with X-Ray wavelength 0.7107 Å (MoKα). Single crystal samples were mounted with Paratone-N acting as an adhesive and cryoprotectant, being cooled to the 150 K by an Oxford Cryosystems COBRA cooler. Collected reflection data was reduced with CryAlisPro (version 171.40.53), The independent atomic model (IAM) solved with using intrinsic phasing using SHELX-T within WinGX (version 2020.2) [23] and refined with SHELX-L. The final IAM model was prepared for publication with Olex2 (version 1.3)[24] The IAM model was also visualised using the ORTEP (Oak Ridge Thermal Ellipsoid Plot) diagrams using ORTEP-III (Oak Ridge National Laboratory, Oak Ridge, USA).102 Below are standard crystallographic results and full geometric details.

Table S1: Crystal data and structure refinement for **1**.

| | | |
|---|---|---|
| Identification code | **1** | |
| Empirical formula | C22 H20 O4 | |
| Formula weight | 348.38 | |
| Temperature | 293(2) K | |
| Wavelength | 0.71073 Å | |
| Crystal system | Monoclinic | |
| Space group | $P2_1/n$ | |
| Unit cell dimensions | a = 8.8357(2) Å | α = 90°. |
| | b = 6.9567(2) Å | β= 92.121(2)°. |
| | c = 30.7382(7) Å | γ = 90°. |
| Volume | 1888.10(8) $Å^3$ | |
| Z | 4 | |

| | |
|---|---|
| Density (calculated) | 1.226 $Mg/m^3$ |
| Absorption coefficient | 0.084 $mm^{-1}$ |
| F(000) | 736 |
| Crystal size | ? x ? x ? $mm^3$ |
| Theta range for data collection | 2.652 to 26.372°. |
| Index ranges | -11<=h<=11, -8<=k<=8, -38<=l<=38 |
| Reflections collected | 29563 |
| Independent reflections | 3849 [R(int) = 0.0290] |
| Completeness to theta = 25.242° | 99.9 % |
| Refinement method | Full-matrix least-squares on $F^2$ |
| Data / restraints / parameters | 3849 / 0 / 349 |
| Goodness-of-fit on $F^2$ | 1.078 |
| Final R indices [I>2sigma(I)] | R1 = 0.0394, wR2 = 0.1057 |
| R indices (all data) | R1 = 0.0443, wR2 = 0.1098 |
| Radiation | MoKα |
| Extinction coefficient | n/a |
| Largest diff. peak and hole | 0.268 and -0.192 e.$Å^{-3}$ |

Table S2: Atomic coordinates ( x $10^4$) and equivalent isotropic displacement parameters ($Å^2$x $10^3$) for **1**. U(eq) is defined as one third of the trace of the orthogonalized $U^{ij}$ tensor.

| | x | y | z | U(eq) |
|---|---|---|---|---|
| C(1) | 5267(1) | 8361(2) | 2797(1) | 22(1) |
| C(2) | 6107(1) | 6848(2) | 2966(1) | 23(1) |
| C(3) | 6279(2) | 5011(2) | 2708(1) | 27(1) |
| C(4) | 5936(2) | 5319(2) | 2225(1) | 28(1) |
| C(5) | 4434(2) | 6348(2) | 2147(1) | 26(1) |
| C(6) | 4431(1) | 8270(2) | 2374(1) | 23(1) |
| C(7) | 6841(1) | 7035(2) | 3389(1) | 24(1) |
| C(8) | 7668(2) | 5399(2) | 3581(1) | 28(1) |
| C(9) | 8593(2) | 5558(2) | 3932(1) | 29(1) |
| C(10) | 3771(1) | 9877(2) | 2213(1) | 25(1) |
| C(1') | 2859(1) | 10110(2) | 1808(1) | 24(1) |
| C(2') | 2847(2) | 11906(2) | 1601(1) | 28(1) |
| C(3') | 2055(2) | 12207(2) | 1211(1) | 29(1) |
| C(4') | 1238(1) | 10713(2) | 1020(1) | 26(1) |
| C(5') | 1166(2) | 8943(2) | 1228(1) | 26(1) |
| C(6') | 1971(1) | 8654(2) | 1617(1) | 26(1) |
| C(1'') | 9496(2) | 4055(2) | 4144(1) | 28(1) |
| C(2'') | 9400(2) | 2116(2) | 4022(1) | 31(1) |
| C(3'') | 10263(2) | 739(2) | 4234(1) | 34(1) |
| C(4'') | 11249(2) | 1252(2) | 4578(1) | 30(1) |
| C(5'') | 11384(2) | 3163(2) | 4701(1) | 31(1) |
| C(6'') | 10517(2) | 4535(2) | 4484(1) | 30(1) |
| O(1) | 5156(1) | 10014(1) | 3013(1) | 28(1) |
| O(2) | 6802(1) | 8621(1) | 3598(1) | 29(1) |
| O(3) | 507(1) | 11043(2) | 627(1) | 32(1) |
| O(4) | 12056(1) | -175(2) | 4781(1) | 43(1) |
| O(1W) | 6329(1) | 9350(2) | 4462(1) | 37(1) |
| O(2W) | -835(2) | 8086(2) | 270(1) | 70(1) |

Table S3: Bond lengths [Å] and angles [°] for **1**.

| | |
|---|---|
| C(1)-O(1) | 1.3333(15) |
| C(1)-C(2) | 1.3792(18) |
| C(1)-C(6) | 1.4717(17) |
| C(2)-C(7) | 1.4364(17) |
| C(2)-C(3) | 1.5155(17) |
| C(3)-C(4) | 1.5189(18) |
| C(3)-H(3A) | 0.974(17) |
| C(3)-H(3B) | 0.976(15) |
| C(4)-C(5) | 1.5192(18) |
| C(4)-H(4A) | 0.989(16) |
| C(4)-H(4B) | 0.990(17) |
| C(5)-C(6) | 1.5089(17) |
| C(5)-H(5A) | 0.985(16) |
| C(5)-H(5B) | 0.972(16) |
| C(6)-C(10) | 1.3469(18) |
| C(7)-O(2) | 1.2772(15) |
| C(7)-C(8) | 1.4642(18) |
| C(8)-C(9) | 1.3358(19) |
| C(8)-H(8) | 0.975(18) |
| C(9)-C(1'') | 1.4536(19) |
| C(9)-H(9) | 0.970(18) |
| C(10)-C(1') | 1.4660(18) |
| C(10)-H(10) | 0.975(17) |
| C(1')-C(6') | 1.3971(18) |
| C(1')-C(2') | 1.4022(18) |
| C(2')-C(3') | 1.3826(18) |
| C(2')-H(2') | 0.985(17) |
| C(3')-C(4') | 1.3832(19) |
| C(3')-H(3') | 0.981(17) |
| C(4')-O(3) | 1.3667(15) |
| C(4')-C(5') | 1.3895(18) |
| C(5')-C(6') | 1.3844(18) |
| C(5')-H(5') | 0.967(15) |
| C(6')-H(6') | 0.951(16) |
| C(1'')-C(6'') | 1.3956(19) |

| | |
|---|---|
| C(1'')-C(2'') | 1.4015(19) |
| C(2'')-C(3'') | 1.374(2) |
| C(2'')-H(2'') | 0.995(17) |
| C(3'')-C(4'') | 1.391(2) |
| C(3'')-H(3'') | 0.962(17) |
| C(4'')-O(4) | 1.3598(17) |
| C(4'')-C(5'') | 1.387(2) |
| C(5'')-C(6'') | 1.3809(19) |
| C(5'')-H(5'') | 1.007(17) |
| C(6'')-H(6'') | 0.994(17) |
| O(1)-H(1) | 0.96(2) |
| O(3)-H(3) | 0.89(2) |
| O(4)-H(4) | 0.96(2) |
| O(1W)-H(1WA) | 0.88(2) |
| O(1W)-H(1WB) | 0.91(3) |
| O(2W)-H(2WA) | 0.88(3) |
| O(2W)-H(2WB) | 0.83(3) |

| | |
|---|---|
| O(1)-C(1)-C(2) | 121.24(11) |
| O(1)-C(1)-C(6) | 115.65(11) |
| C(2)-C(1)-C(6) | 123.11(11) |
| C(1)-C(2)-C(7) | 119.41(11) |
| C(1)-C(2)-C(3) | 120.57(11) |
| C(7)-C(2)-C(3) | 120.01(11) |
| C(2)-C(3)-C(4) | 111.90(11) |
| C(2)-C(3)-H(3A) | 110.7(9) |
| C(4)-C(3)-H(3A) | 108.9(9) |
| C(2)-C(3)-H(3B) | 110.6(8) |
| C(4)-C(3)-H(3B) | 108.3(8) |
| H(3A)-C(3)-H(3B) | 106.2(12) |
| C(3)-C(4)-C(5) | 111.22(11) |
| C(3)-C(4)-H(4A) | 109.0(9) |
| C(5)-C(4)-H(4A) | 109.5(9) |
| C(3)-C(4)-H(4B) | 110.2(9) |
| C(5)-C(4)-H(4B) | 109.7(9) |
| H(4A)-C(4)-H(4B) | 107.1(13) |
| C(6)-C(5)-C(4) | 111.16(10) |

| | |
|---|---|
| C(6)-C(5)-H(5A) | 110.0(9) |
| C(4)-C(5)-H(5A) | 109.5(9) |
| C(6)-C(5)-H(5B) | 111.0(9) |
| C(4)-C(5)-H(5B) | 108.7(9) |
| H(5A)-C(5)-H(5B) | 106.3(13) |
| C(10)-C(6)-C(1) | 119.08(11) |
| C(10)-C(6)-C(5) | 124.98(11) |
| C(1)-C(6)-C(5) | 115.91(11) |
| O(2)-C(7)-C(2) | 120.87(11) |
| O(2)-C(7)-C(8) | 119.46(11) |
| C(2)-C(7)-C(8) | 119.66(11) |
| C(9)-C(8)-C(7) | 122.85(13) |
| C(9)-C(8)-H(8) | 121.7(10) |
| C(7)-C(8)-H(8) | 115.4(10) |
| C(8)-C(9)-C(1'') | 127.72(13) |
| C(8)-C(9)-H(9) | 117.2(11) |
| C(1'')-C(9)-H(9) | 115.1(11) |
| C(6)-C(10)-C(1') | 128.41(12) |
| C(6)-C(10)-H(10) | 117.0(9) |
| C(1')-C(10)-H(10) | 114.5(9) |
| C(6')-C(1')-C(2') | 117.41(11) |
| C(6')-C(1')-C(10) | 123.99(12) |
| C(2')-C(1')-C(10) | 118.59(11) |
| C(3')-C(2')-C(1') | 121.60(12) |
| C(3')-C(2')-H(2') | 118.0(9) |
| C(1')-C(2')-H(2') | 120.4(9) |
| C(2')-C(3')-C(4') | 119.58(12) |
| C(2')-C(3')-H(3') | 121.6(10) |
| C(4')-C(3')-H(3') | 118.9(10) |
| O(3)-C(4')-C(3') | 118.03(12) |
| O(3)-C(4')-C(5') | 121.75(12) |
| C(3')-C(4')-C(5') | 120.21(12) |
| C(6')-C(5')-C(4') | 119.67(12) |
| C(6')-C(5')-H(5') | 119.7(9) |
| C(4')-C(5')-H(5') | 120.6(9) |
| C(5')-C(6')-C(1') | 121.40(12) |
| C(5')-C(6')-H(6') | 117.4(9) |

| | |
|---|---|
| C(1')-C(6')-H(6') | 121.2(9) |
| C(6")-C(1")-C(2") | 117.49(12) |
| C(6")-C(1")-C(9) | 119.43(12) |
| C(2")-C(1")-C(9) | 123.08(12) |
| C(3")-C(2")-C(1") | 121.15(13) |
| C(3")-C(2")-H(2") | 118.4(9) |
| C(1")-C(2")-H(2") | 120.4(9) |
| C(2")-C(3")-C(4") | 120.17(13) |
| C(2")-C(3")-H(3") | 122.7(10) |
| C(4")-C(3")-H(3") | 117.1(10) |
| O(4)-C(4")-C(5") | 122.46(12) |
| O(4)-C(4")-C(3") | 117.64(13) |
| C(5")-C(4")-C(3") | 119.90(13) |
| C(6")-C(5")-C(4") | 119.37(13) |
| C(6")-C(5")-H(5") | 122.0(10) |
| C(4")-C(5")-H(5") | 118.7(10) |
| C(5")-C(6")-C(1") | 121.90(13) |
| C(5")-C(6")-H(6") | 120.5(9) |
| C(1")-C(6")-H(6") | 117.6(9) |
| C(1)-O(1)-H(1) | 104.3(13) |
| C(4')-O(3)-H(3) | 110.4(13) |
| C(4")-O(4)-H(4) | 108.2(14) |
| H(1WA)-O(1W)-H(1WB) | 99.8(19) |
| H(2WA)-O(2W)-H(2WB) | 109(2) |

Symmetry transformations used to generate equivalent atoms:

Table S4: Anisotropic displacement parameters (Å$^2$x 10$^3$)for **1**. The anisotropic displacement factor exponent takes the form: -2p$^2$[ h$^2$a*$^2$U$^{11}$ + ... + 2 h k a* b* U$^{12}$ ]

| | $U^{11}$ | $U^{22}$ | $U^{33}$ | $U^{23}$ | $U^{13}$ | $U^{12}$ |
|---|---|---|---|---|---|---|
| C(1) | 23(1) | 23(1) | 21(1) | -3(1) | 3(1) | -3(1) |
| C(2) | 23(1) | 25(1) | 22(1) | -1(1) | 1(1) | -1(1) |

| | | | | | | |
|---|---|---|---|---|---|---|
| C(3) | 31(1) | 25(1) | 26(1) | -3(1) | -2(1) | 5(1) |
| C(4) | 29(1) | 28(1) | 25(1) | -7(1) | 0(1) | 4(1) |
| C(5) | 28(1) | 26(1) | 23(1) | -4(1) | -2(1) | 1(1) |
| C(6) | 22(1) | 26(1) | 22(1) | -2(1) | 1(1) | -1(1) |
| C(7) | 25(1) | 26(1) | 22(1) | 0(1) | 2(1) | -3(1) |
| C(8) | 31(1) | 28(1) | 25(1) | 0(1) | -1(1) | 0(1) |
| C(9) | 31(1) | 28(1) | 29(1) | -1(1) | -1(1) | -1(1) |
| C(10) | 26(1) | 26(1) | 23(1) | -3(1) | 1(1) | -1(1) |
| C(1') | 24(1) | 27(1) | 23(1) | 0(1) | 1(1) | 2(1) |
| C(2') | 30(1) | 24(1) | 28(1) | -3(1) | -3(1) | -1(1) |
| C(3') | 35(1) | 23(1) | 28(1) | 3(1) | -2(1) | 0(1) |
| C(4') | 27(1) | 29(1) | 21(1) | 1(1) | -2(1) | 2(1) |
| C(5') | 27(1) | 25(1) | 27(1) | -1(1) | -3(1) | -3(1) |
| C(6') | 27(1) | 24(1) | 26(1) | 4(1) | 0(1) | 0(1) |
| C(1'') | 28(1) | 31(1) | 24(1) | 1(1) | 1(1) | 0(1) |
| C(2'') | 33(1) | 32(1) | 28(1) | -1(1) | -4(1) | -2(1) |
| C(3'') | 41(1) | 27(1) | 33(1) | -1(1) | -3(1) | 0(1) |
| C(4'') | 35(1) | 30(1) | 26(1) | 2(1) | 0(1) | 5(1) |
| C(5'') | 34(1) | 34(1) | 25(1) | -2(1) | -4(1) | 1(1) |
| C(6'') | 34(1) | 29(1) | 27(1) | -2(1) | -2(1) | 0(1) |
| O(1) | 35(1) | 24(1) | 24(1) | -5(1) | -4(1) | 3(1) |
| O(2) | 36(1) | 28(1) | 22(1) | -2(1) | -4(1) | 0(1) |
| O(3) | 42(1) | 29(1) | 25(1) | 4(1) | -9(1) | -3(1) |
| O(4) | 58(1) | 34(1) | 37(1) | -1(1) | -15(1) | 11(1) |
| O(1W) | 46(1) | 37(1) | 26(1) | -4(1) | -2(1) | -3(1) |
| O(2W) | 119(1) | 57(1) | 31(1) | 11(1) | -26(1) | -50(1) |

Table S5: Hydrogen coordinates ( x $10^4$) and isotropic displacement parameters ($Å^2$x $10^3$) for **1**.

| | x | y | z | U(eq) |
|---|---|---|---|---|
| H(1WA) | 6620(20) | 9180(30) | 4193(8) | 64(6) |
| H(2WA) | -960(30) | 6910(40) | 371(8) | 71(7) |
| H(1WB) | 5640(30) | 8390(30) | 4479(7) | 71(7) |

| | | | | |
|---|---|---|---|---|
| H(2WB) | -1210(30) | 8140(30) | 20(8) | 75(7) |
| H(3A) | 5612(18) | 4010(20) | 2813(5) | 31(4) |
| H(3B) | 7307(17) | 4510(20) | 2745(5) | 22(3) |
| H(4A) | 6758(18) | 6090(20) | 2103(5) | 30(4) |
| H(4B) | 5916(18) | 4070(20) | 2070(5) | 37(4) |
| H(5A) | 4238(17) | 6520(20) | 1832(5) | 34(4) |
| H(5B) | 3627(17) | 5530(20) | 2248(5) | 28(4) |
| H(1) | 5740(20) | 9810(30) | 3278(7) | 68(6) |
| H(3) | 40(20) | 9980(30) | 531(7) | 58(6) |
| H(4) | 12580(30) | 360(30) | 5032(8) | 73(7) |
| H(8) | 7516(19) | 4180(30) | 3430(6) | 43(5) |
| H(9) | 8700(20) | 6820(30) | 4063(6) | 45(5) |
| H(10) | 3948(17) | 11060(20) | 2376(5) | 33(4) |
| H(2') | 3427(18) | 12990(20) | 1729(5) | 34(4) |
| H(3') | 2068(19) | 13460(20) | 1062(6) | 40(4) |
| H(5') | 575(17) | 7900(20) | 1100(5) | 27(4) |
| H(6') | 1883(17) | 7430(20) | 1752(5) | 32(4) |
| H(2'') | 8672(18) | 1700(20) | 3786(5) | 35(4) |
| H(3'') | 10187(18) | -610(30) | 4165(5) | 36(4) |
| H(5'') | 12123(18) | 3510(20) | 4946(5) | 36(4) |
| H(6'') | 10594(18) | 5910(20) | 4569(5) | 35(4) |

HPLC report for compound **2**

SHIMADZU LabSolutions **Analysis Report**

**<Sample Information>**

Sample Name : JL-17-3 100ug/mL
Sample ID : JT-17-3
Data Filename : JL-17-3 100ugmL 110222.lcd
Method Filename : laith.lcm
Batch Filename : JT_JL batch 110222.lcb
Vial # : 1-3
Injection Volume : 10 uL
Date Acquired : 11/02/2022 11:15:38 AM
Date Processed : 11/02/2022 11:35:42 AM

Sample Type : Standard
Level : 1
Acquired by : System Administrator
Processed by : System Administrator

**<Chromatogram>**

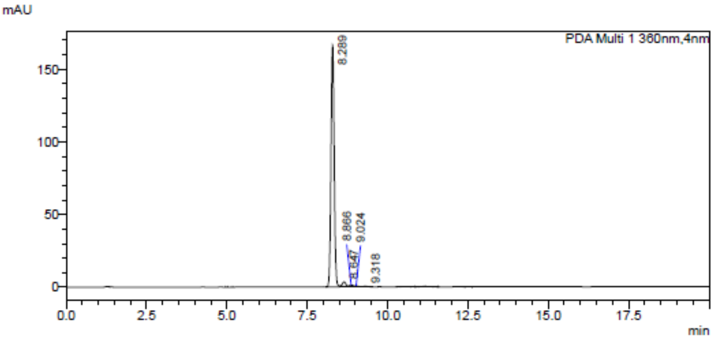


**<Peak Table>**

PDA Ch1 360nm

| Peak# | Ret. Time | Area | Height | Conc. | Unit | Mark | Name |
|---|---|---|---|---|---|---|---|
| 1 | 8.289 | 1144782 | 165814 | 96.056 | | | |
| 2 | 8.647 | 27763 | 3426 | 2.330 | | V | |
| 3 | 8.866 | 10630 | 1419 | 0.892 | | V | |
| 4 | 9.024 | 5148 | 714 | 0.432 | | V | |
| 5 | 9.318 | 3462 | 358 | 0.291 | | V | |
| Total | | 1191786 | 171731 | | | | |

HPLC report for compound **3**

SHIMADZU LabSolutions **Analysis Report**

**<Sample Information>**

Sample Name : JT-305 100ug/mL
Sample ID : JT-305
Data Filename : JT-305 100ugmL 110222.lcd
Method Filename : laith.lcm
Batch Filename : JT_JL batch 110222.lcb
Vial # : 1-1
Injection Volume : 10 uL
Date Acquired : 11/02/2022 10:55:05 AM
Date Processed : 11/02/2022 11:15:09 AM

Sample Type : Standard
Level : 1
Acquired by : System Administrator
Processed by : System Administrator

**<Chromatogram>**

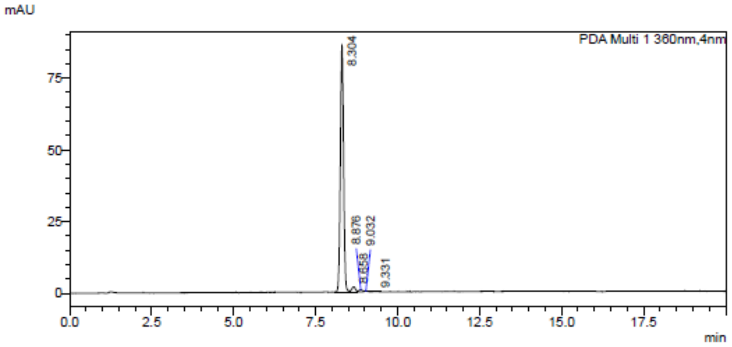


**<Peak Table>**

PDA Ch1 360nm

| Peak# | Ret. Time | Area | Height | Conc. | Unit | Mark | Name |
|---|---|---|---|---|---|---|---|
| 1 | 8.304 | 590174 | 85637 | 96.238 | | | |
| 2 | 8.658 | 13997 | 1759 | 2.282 | | V | |
| 3 | 8.876 | 5325 | 700 | 0.868 | | V | |
| 4 | 9.032 | 2265 | 344 | 0.369 | | V | |
| 5 | 9.331 | 1481 | 174 | 0.241 | | V | |
| Total | | 613241 | 88615 | | | | |

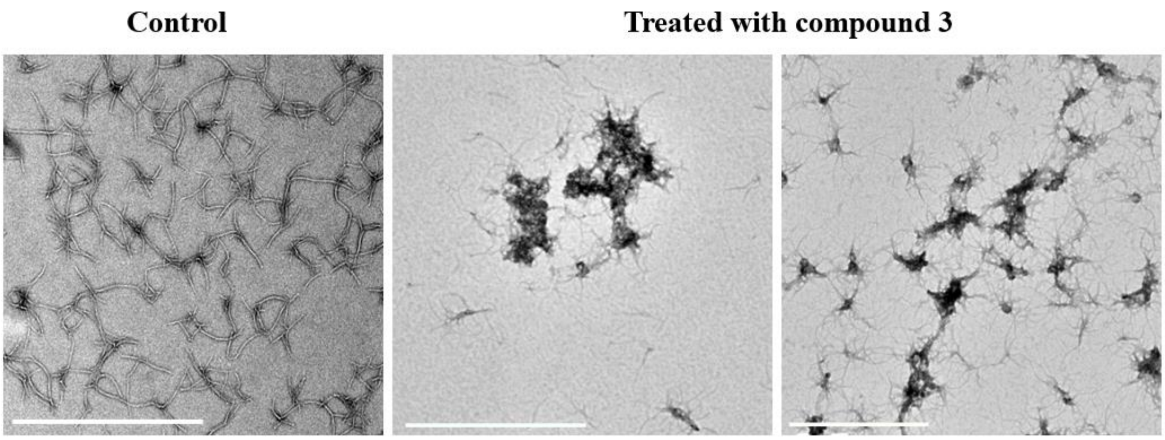


**Figure S1** Compound 3 causes aggregation of *Bs*FtsZ. Transmission Electron Microscopy images of *Bacillus subtilis* FtsZ (*Bs*FtsZ) filaments formed in the presence and absence of **3**. *Bs*FtsZ (5 μM) in PKM buffer was incubated without and with 20 μM **3** for 20 minutes at 25 °C before polymerizing in the presence of 1 mM GTP. Scale bar indicates 1 μm. This experiment was performed three times.

**References**

23. Farrugia, L. J., WinGX suite for small-molecule single-crystal crystallography. *J. Appl. Cryst*., **1999**, 32(4), 837-838.

24. Dolomanov, O. V.; Bourhis, L. J.; Gildea, R. J.; Howard, J. A. K.; Puschmann, H., OLEX2: a complete structure solution, refinement and analysis program. *J. Appl. Cryst.* **2009**, 42(2), 339-341.
